# Supplementary material for: Deletion of wfs1 Impairs Oligodendrocyte Precursor Cells Dorsal Distribution and Myelination Through the wfs1-hmgcs1 Axis in Zebrafish
Source: Biology (Basel). 2026 Aug 16;15(16):1402. doi: 10.3390/biology15161402 (PMC13510151; doi:10.3390/biology15161402)
Supplement: Supplementary file 1 [file biology-15-01402-s001.zip › File S1-Transcriptome sequencing data and differentially expressed genes (DEGs) in wfs1-- zebrafish at 5 dpf/2.KEGG/wfs1KOvsWT/GSEA/WT/ERBB_SIGNALING_PATHWAY/ERBB_SIGNALING_PATHWAY(DRE04012).html]

Details for gene set ERBB\_SIGNALING\_PATHWAY(DRE04012)[GSEA]

|  || Dataset | deg.sample |
| Phenotype | sample.cls |
| Upregulated in class | WT |
| GeneSet | ERBB\_SIGNALING\_PATHWAY(DRE04012) |
| Enrichment Score (ES) | -0.218991 |
| Normalized Enrichment Score (NES) | -0.9794126 |
| Nominal p-value | 0.49323016 |
| FDR q-value | 0.6355439 |
| FWER p-Value | 1.0 |
Table: GSEA Results Summary

  

Fig 1: Enrichment plot: ERBB\_SIGNALING\_PATHWAY(DRE04012)      
 Profile of the Running ES Score & Positions of GeneSet Members on the Rank Ordered List

  

| PROBE | DESCRIPTION (from dataset) | GENE SYMBOL | GENE\_TITLE | RANK IN GENE LIST | RANK METRIC SCORE | RUNNING ES | CORE ENRICHMENT || 1 | 544664\_1 | erbb3b |  |  | 580 | 2.789 | 0.0219 | No |
| 2 | 100006675 | prkcba |  |  | 711 | 2.563 | 0.0547 | No |
| 3 | 100000252\_3 | pik3r3b |  |  | 716 | 2.550 | 0.0913 | No |
| 4 | 556277 | cblb |  |  | 1038 | 2.056 | 0.1107 | No |
| 5 | 100151416 | cdkn1a |  |  | 1627 | 1.584 | 0.1150 | No |
| 6 | 100000252 | pik3r3b |  |  | 1965 | 1.382 | 0.1243 | No |
| 7 | 404211 | pik3r2 |  |  | 4059 | 0.817 | 0.0702 | No |
| 8 | 556277\_1 | cblb |  |  | 4343 | 0.770 | 0.0724 | No |
| 9 | 101882495\_1 | nrg3b |  |  | 4392 | 0.763 | 0.0818 | No |
| 10 | 562011\_1 | shc2 |  |  | 4943 | 0.688 | 0.0744 | No |
| 11 | 100270726\_1 | nrg2b |  |  | 5219 | 0.653 | 0.0752 | No |
| 12 | 562242\_1 | rps6kb1a |  |  | 5344 | 0.642 | 0.0805 | No |
| 13 | 564921 | bada |  |  | 5627 | 0.623 | 0.0806 | No |
| 14 | 561747 | plcg2 |  |  | 5761 | 0.610 | 0.0852 | No |
| 15 | 58100 | badb |  |  | 5892 | 0.598 | 0.0897 | No |
| 16 | 570636 | abl2 |  |  | 5926 | 0.595 | 0.0972 | No |
| 17 | 386635 | erbb3a |  |  | 5954 | 0.592 | 0.1049 | No |
| 18 | 100007677 | erbb4a |  |  | 6125 | 0.574 | 0.1078 | No |
| 19 | 797938 | hbegfa |  |  | 6257 | 0.561 | 0.1118 | No |
| 20 | 798222 | camk2b |  |  | 6413 | 0.544 | 0.1147 | No |
| 21 | 567895 | elk1 |  |  | 6507 | 0.536 | 0.1195 | No |
| 22 | 30654 | gsk3b |  |  | 6859 | 0.504 | 0.1157 | No |
| 23 | 325084 | src |  |  | 6875 | 0.502 | 0.1225 | No |
| 24 | 30686 | myca |  |  | 6877 | 0.502 | 0.1297 | No |
| 25 | 65236 | mapk8b |  |  | 6998 | 0.492 | 0.1330 | No |
| 26 | 561737 | pik3ca |  |  | 7195 | 0.474 | 0.1336 | No |
| 27 | 335916 | jun |  |  | 7293 | 0.467 | 0.1373 | No |
| 28 | 793359\_1 | camk2g2 |  |  | 7602 | 0.447 | 0.1340 | No |
| 29 | 796461 | nrg1 |  |  | 7640 | 0.443 | 0.1392 | No |
| 30 | 100000720 | abl1 |  |  | 8525 | 0.401 | 0.1172 | No |
| 31 | 393972 | nck2a |  |  | 8571 | 0.398 | 0.1215 | No |
| 32 | 556443 | crkl |  |  | 8881 | 0.376 | 0.1172 | No |
| 33 | 560913 | map2k7 |  |  | 9023 | 0.366 | 0.1180 | No |
| 34 | 324254 | mtor |  |  | 9164 | 0.355 | 0.1187 | No |
| 35 | 378972 | akt2 |  |  | 9293 | 0.347 | 0.1197 | No |
| 36 | 793359 | camk2g2 |  |  | 9631 | 0.325 | 0.1137 | No |
| 37 | 373103 | pak1 |  |  | 9754 | 0.318 | 0.1145 | No |
| 38 | 562720 | mapk8a |  |  | 9888 | 0.310 | 0.1148 | No |
| 39 | 492364 | cbl |  |  | 9938 | 0.307 | 0.1176 | No |
| 40 | 100006951 | nrg2a |  |  | 10108 | 0.295 | 0.1166 | No |
| 41 | 568951 | map2k4b |  |  | 10345 | 0.282 | 0.1132 | No |
| 42 | 569836 | erbb4b |  |  | 10364 | 0.281 | 0.1167 | No |
| 43 | 393141 | mycb |  |  | 10475 | 0.274 | 0.1172 | No |
| 44 | 393228\_1 | zgc:55558 |  |  | 10754 | 0.257 | 0.1121 | No |
| 45 | 563181 | map2k2a |  |  | 10792 | 0.256 | 0.1146 | No |
| 46 | 497384 | prkca |  |  | 10900 | 0.249 | 0.1148 | No |
| 47 | 503869 | btc |  |  | 10988 | 0.244 | 0.1156 | No |
| 48 | 403052 | map2k4a |  |  | 11173 | 0.234 | 0.1132 | No |
| 49 | 100034402 | tgfa |  |  | 11448 | 0.219 | 0.1077 | No |
| 50 | 393953 | prkcbb |  |  | 11901 | 0.196 | 0.0963 | No |
| 51 | 101885162 | si:ch73-383l1.1 |  |  | 12003 | 0.191 | 0.0959 | No |
| 52 | 799566\_1 | eif4ebp1 |  |  | 12044 | 0.188 | 0.0973 | No |
| 53 | 100003514 | prkcg |  |  | 12176 | 0.181 | 0.0958 | No |
| 54 | 567874 | pak6b |  |  | 12305 | 0.174 | 0.0943 | No |
| 55 | 368329 | cdkn1bb |  |  | 12428 | 0.168 | 0.0929 | No |
| 56 | 402862 | cdkn1ba |  |  | 12928 | 0.144 | 0.0792 | No |
| 57 | 562064 | camk2b1 |  |  | 13117 | 0.134 | 0.0752 | No |
| 58 | 100535494\_1 | LOC100535494 |  |  | 13184 | 0.131 | 0.0750 | No |
| 59 | 569698 | mapk10 |  |  | 13907 | 0.096 | 0.0537 | No |
| 60 | 561670 | sos2 |  |  | 14111 | 0.087 | 0.0486 | No |
| 61 | 100334953 | nck1b |  |  | 14168 | 0.084 | 0.0480 | No |
| 62 | 100149794 | akt3b |  |  | 14249 | 0.080 | 0.0466 | No |
| 63 | 100535494 | LOC100535494 |  |  | 14438 | 0.073 | 0.0418 | No |
| 64 | 323818 | shc1 |  |  | 14931 | 0.050 | 0.0270 | No |
| 65 | 30380 | nras |  |  | 15635 | 0.015 | 0.0051 | No |
| 66 | 406308 | grb2b |  |  | 15876 | 0.003 | -0.0024 | No |
| 67 | 445474 | stat5b |  |  | 15905 | 0.002 | -0.0033 | No |
| 68 | 373867 | plcg1 |  |  | 16057 | -0.004 | -0.0080 | No |
| 69 | 791141 | zgc:158659 |  |  | 16140 | -0.006 | -0.0105 | No |
| 70 | 378478 | egfra |  |  | 16429 | -0.015 | -0.0193 | No |
| 71 | 405898 | pak7 |  |  | 17152 | -0.039 | -0.0415 | No |
| 72 | 100149273 | mapk9 |  |  | 17865 | -0.070 | -0.0629 | No |
| 73 | 394174 | pik3cd |  |  | 17974 | -0.074 | -0.0652 | No |
| 74 | 431769 | pak4 |  |  | 17988 | -0.075 | -0.0646 | No |
| 75 | 142986 | ptk2ab |  |  | 18232 | -0.087 | -0.0710 | No |
| 76 | 445098 | nck2b |  |  | 18379 | -0.093 | -0.0742 | No |
| 77 | 404211\_1 | pik3r2 |  |  | 18555 | -0.101 | -0.0783 | No |
| 78 | 386966 | erbb2 |  |  | 18881 | -0.117 | -0.0868 | No |
| 79 | 560549 | akt3a |  |  | 19324 | -0.136 | -0.0988 | No |
| 80 | 399480 | mapk3 |  |  | 19514 | -0.144 | -0.1026 | No |
| 81 | 406728 | map2k1 |  |  | 19540 | -0.145 | -0.1013 | No |
| 82 | 445234\_2 | crk |  |  | 19651 | -0.150 | -0.1026 | No |
| 83 | 406349\_1 | rps6kb1b |  |  | 19701 | -0.153 | -0.1020 | No |
| 84 | 557109 | raf1b |  |  | 20203 | -0.175 | -0.1152 | No |
| 85 | 791855 | camk2g1 |  |  | 20264 | -0.177 | -0.1146 | No |
| 86 | 407664 | hbegfb |  |  | 20560 | -0.191 | -0.1211 | No |
| 87 | 493609\_1 | grb2a |  |  | 20689 | -0.198 | -0.1223 | No |
| 88 | 325034 | pak2b |  |  | 20929 | -0.212 | -0.1268 | No |
| 89 | 564584 | nck1a |  |  | 21322 | -0.232 | -0.1358 | No |
| 90 | 503869\_1 | btc |  |  | 21782 | -0.256 | -0.1465 | No |
| 91 | 100332111 | shc3 |  |  | 21977 | -0.266 | -0.1488 | No |
| 92 | 550436 | camk2a |  |  | 22386 | -0.289 | -0.1575 | No |
| 93 | 324254\_1 | mtor |  |  | 22584 | -0.301 | -0.1594 | No |
| 94 | 403065 | braf |  |  | 22710 | -0.308 | -0.1589 | No |
| 95 | 266749 | pak2a |  |  | 23101 | -0.334 | -0.1663 | No |
| 96 | 360144\_1 | mapk1 |  |  | 23446 | -0.357 | -0.1720 | No |
| 97 | 100002263 | pik3cb |  |  | 23508 | -0.361 | -0.1688 | No |
| 98 | 557882 | si:dkeyp-80c12.7 |  |  | 23526 | -0.363 | -0.1641 | No |
| 99 | 557176 | pik3r1 |  |  | 23610 | -0.367 | -0.1614 | No |
| 100 | 369197\_1 | stat5a |  |  | 23647 | -0.370 | -0.1572 | No |
| 101 | 100536197 | LOC100536197 |  |  | 24189 | -0.406 | -0.1684 | No |
| 102 | 100002225 | map2k2b |  |  | 24736 | -0.433 | -0.1793 | No |
| 103 | 436815 | camk2d2 |  |  | 25880 | -0.518 | -0.2079 | No |
| 104 | 30716 | raf1a |  |  | 25882 | -0.518 | -0.2004 | No |
| 105 | 550286 | hrasa |  |  | 26472 | -0.575 | -0.2107 | Yes |
| 106 | 403063 | araf |  |  | 26515 | -0.580 | -0.2037 | Yes |
| 107 | 100333967 | si:ch73-374l24.1 |  |  | 26564 | -0.584 | -0.1968 | Yes |
| 108 | 445208 | camk2d1 |  |  | 26863 | -0.615 | -0.1973 | Yes |
| 109 | 386705 | ptk2aa |  |  | 26978 | -0.627 | -0.1919 | Yes |
| 110 | 406666 | gab1 |  |  | 27046 | -0.633 | -0.1849 | Yes |
| 111 | 101882495 | nrg3b |  |  | 27312 | -0.650 | -0.1839 | Yes |
| 112 | 553656 | hrasb |  |  | 27554 | -0.680 | -0.1817 | Yes |
| 113 | 100270726 | nrg2b |  |  | 27799 | -0.716 | -0.1790 | Yes |
| 114 | 497384\_1 | prkca |  |  | 27854 | -0.723 | -0.1703 | Yes |
| 115 | 403045\_1 | egf |  |  | 28202 | -0.773 | -0.1702 | Yes |
| 116 | 101910198 | akt1 |  |  | 28441 | -0.810 | -0.1660 | Yes |
| 117 | 445289 | kras |  |  | 29269 | -0.994 | -0.1777 | Yes |
| 118 | 403146 | akt2l |  |  | 29342 | -1.012 | -0.1654 | Yes |
| 119 | 556286 | sos1 |  |  | 29488 | -1.060 | -0.1547 | Yes |
| 120 | 369197 | stat5a |  |  | 29591 | -1.090 | -0.1422 | Yes |
| 121 | 100006675\_1 | prkcba |  |  | 30078 | -1.276 | -0.1392 | Yes |
| 122 | 799566 | eif4ebp1 |  |  | 30170 | -1.323 | -0.1230 | Yes |
| 123 | 556277\_2 | cblb |  |  | 30334 | -1.406 | -0.1079 | Yes |
| 124 | 58100\_1 | badb |  |  | 31292 | -2.420 | -0.1032 | Yes |
| 125 | 571431 | LOC571431 |  |  | 31422 | -2.716 | -0.0682 | Yes |
| 126 | 100000252\_1 | pik3r3b |  |  | 31434 | -2.733 | -0.0292 | Yes |
| 127 | 544664 | erbb3b |  |  | 31538 | -3.021 | 0.0111 | Yes |
Table: GSEA details [plain text format]

  

Fig 2: ERBB\_SIGNALING\_PATHWAY(DRE04012)      
 Blue-Pink O' Gram in the Space of the Analyzed GeneSet

  

Fig 3: ERBB\_SIGNALING\_PATHWAY(DRE04012): Random ES distribution      
 Gene set null distribution of ES for **ERBB\_SIGNALING\_PATHWAY(DRE04012)**
